# Long term follow-up of immediately temporized zirconia and titanium one-piece dental implants–a prospective cohort study

**DOI:** 10.1186/s40729-025-00655-1

**Published:** 2025-12-02

**Authors:** Valentin Herber, Elisabeth Steyer, Martin Koller, Yalda Nassehi, Anke Pichler, Michael Payer

**Affiliations:** 1 Department of Dentistry and Oral Health, Division of Oral Surgery and Orthodontics, Medical University of Graz, Billrothgasse 4, 8010 Graz, Austria; 2 Department of Oral Surgery, University Center for Dental Medicine, University of Basel, Mattenstrasse 40, 4058 Basel, Switzerland; 3 Department of Dentistry and Oral Health, Division of Restorative Dentistry, Periodontology and Prosthodontics, Medical University of Graz, Billrothgasse 4, 8010 Graz, Austria

**Keywords:** All-ceramic crowns, Titanium implants, Yttria-stabilised zirconia, One-piece implants

## Abstract

**Objectives:**

This prospective cohort study aimed to evaluate the clinical and radiographic outcomes of one-piece zirconia implants and compare them to those of titanium implants over a period of up to 108 months (85.3 ± 31.6 months).

**Methods:**

A total of 33 implants (22 zirconia/Ziterion Zit-Z® and 11 titanium/Ziterion Zit-T®) were placed in 32 patients (15 male, 17 female), all of whom required neither bone nor soft tissue augmentation. These implant systems are no longer commercially available. Radiographic bone levels, clinical peri-implant parameters, and implant survival were recorded for up to 108 months.

**Results:**

Implant fractures were not observed. In the titanium group, two implant failures (18.2%) were reported, while eight zirconia implants (36.4%) failed. Kaplan–Meier analysis showed survival rates of 80.8 and 62.2% for titanium and zirconia implants, respectively, for up to 108 months. The bleeding on probing values gradually increased over time in both groups. A statistically significant decrease in the plaque index was observed for zirconia implants at 36 and 108 months (*p* < 0.001). Additionally, both groups showed a statistically significant reduction in marginal bone resorption within the first year after implant placement (*p* < 0.05). However, no significant difference between the two groups was observed over time (*p *> 0.05).

**Conclusions:**

Immediately temporized one-piece zirconia implants showed lower survival rates than those of titanium implants, highlighting the need for further validation in larger trials.

**Clinical relevance:**

Immediate temporization of non-commercial zirconia implants is not recommended due to their compromised survival rates.

## Introduction

Over the past several decades, especially since the late 1960s and the first report of dental implants by Per-Ingvar Brånemark, implant dentistry research has gained worldwide interest [1]. The rehabilitation of partially or fully edentulous patients through the replacement of missing teeth with implants and implant-supported prostheses has become routine therapy for clinicians. Medical-grade commercially pure titanium or titanium alloy are commonly used as a material in implant dentistry and is considered the gold standard for the fabrication of dental implants. Titanium implants have shown clinically high survival rates of up to 97.2% with single-crown-supporting implants [2]. Owing to concerns regarding titanium, specifically biological and technical complications, alternative materials have been sought [3]. The release of micro- and nanoscale titanium particles into the surrounding tissues may have damaging effects on intraepithelial homeostasis, potentially leading to implant detachment, as well as promoting bone resorption through the stimulation of osteoclast differentiation [3, 4]. Additionally, wear of metallic dental implants at the time of insertion has been confirmed in vitro [5]. Further literature reviews have showed that continuous long-term corrosion reactions lead to the release of titanium ions into peri-implant tissues, potentially causing biological and technical complications, such as material fatigue [6].

Recently, research on ceramic implants has renewed interest, several years after their first clinical implementation in the 1970s. Ceramic implants made from zirconium dioxide (also known as zirconia) have been proposed as an alternative implant material [7, 8]. The biocompatibility and hard tissue integration of zirconia materials have been validated pre-clinically using different large animal models [9]. Moreover, to optimise the osseointegration of zirconia implants, surface modifications including sandblasting, etching, sintering, and coating have been evaluated [10]. Owing to its tooth-like colour, zirconia implants may offer benefits for patients with thin tissue biotypes or those requiring implantation in a highly aesthetic anterior area [11]. Aesthetic issues may arise from the dull greyish discoloration of titanium, which is not always concealed by the surrounding soft tissues, particularly in cases with a thin tissue biotype [12]. Zirconia has been reported to have a low affinity for oral biofilm which is believed to improve soft tissue integration around the implant [13]. A reduction in bacterial colonization can potentially minimize inflammation and peri-implant tissue damage, leading to better long-term soft tissue stability of the implant. A recent systematic review and meta-analysis also demonstrated comparable survival and success rates between zirconia and titanium implants in short-term follow-up [14].

However, there is a lack of clinical data in the literature regarding zirconia implants with long-term follow-up, particularly those subjected to immediate loading. Therefore, the primary aim of this study was to evaluate the clinical and radiographic outcomes of one-piece zirconia implants and compare them to those of titanium implants over a period of up to 108 months. Based on the aim of this study, the following null hypothesis was formulated: There is no statistically significant difference in clinical and radiographic outcomes between zirconia and titanium one-piece implants restored with lithium disilicate crowns over the observation period. The secondary aim was to evaluate the survival rates of the zirconia and titanium one-piece implants.

## Materials and methods

### Study population

This study was conducted as a prospective, cohort study for the implant rehabilitation of partially edentulous patients, using one-piece yttria-stabilised zirconia implants in comparison with titanium implants. This trial included 32 patients recruited from the dental school at the Medical University of Graz, Austria. The study was approved by the Ethics Committee of the Medical University of Graz (20–340 ex 08/09) and was conducted in accordance with Good Clinical Practice standards and the Declaration of Helsinki.

The inclusion criteria were subjects aged 18 years or older who had provided informed and written consent, with tooth gaps of up to three missing units, sufficient peri-implant soft tissue volume, and adequate horizontal and vertical bone for the placement of implants with a minimum length of 10 mm and a width of 3.5 mm. Additionally, patient acceptance of the scheduled visits for clinical and radiographic analysis, as well as maintenance, was mandatory. Patients with any of the following exclusion criteria were not allowed to participate: (i) lack of compliance or failure to provide consent, (ii) signs of occlusal dysfunction or parafunctions (e.g. bruxers), (iii) current acute periodontal disease, (iv) general contraindications to implant treatment, (v) metabolic skeletal diseases (e.g., osteomalacia or osteoporosis), (vi) medication potentially affecting bone structure and metabolism (such as corticosteroid treatment or bisphosphonate/denosumab therapy), (vii) pregnancy, confirmed through a pregnancy test (HCG Schnelltest, DiaChrom bj-giagnostik, Giessen, Germany), (viii) previous irradiation in the neck/head area, (ix) need for bone and soft tissue augmentation procedures and (x) smokers consuming more than 10 cigarettes per day.

### Clinical procedures

**Fig. 1:**
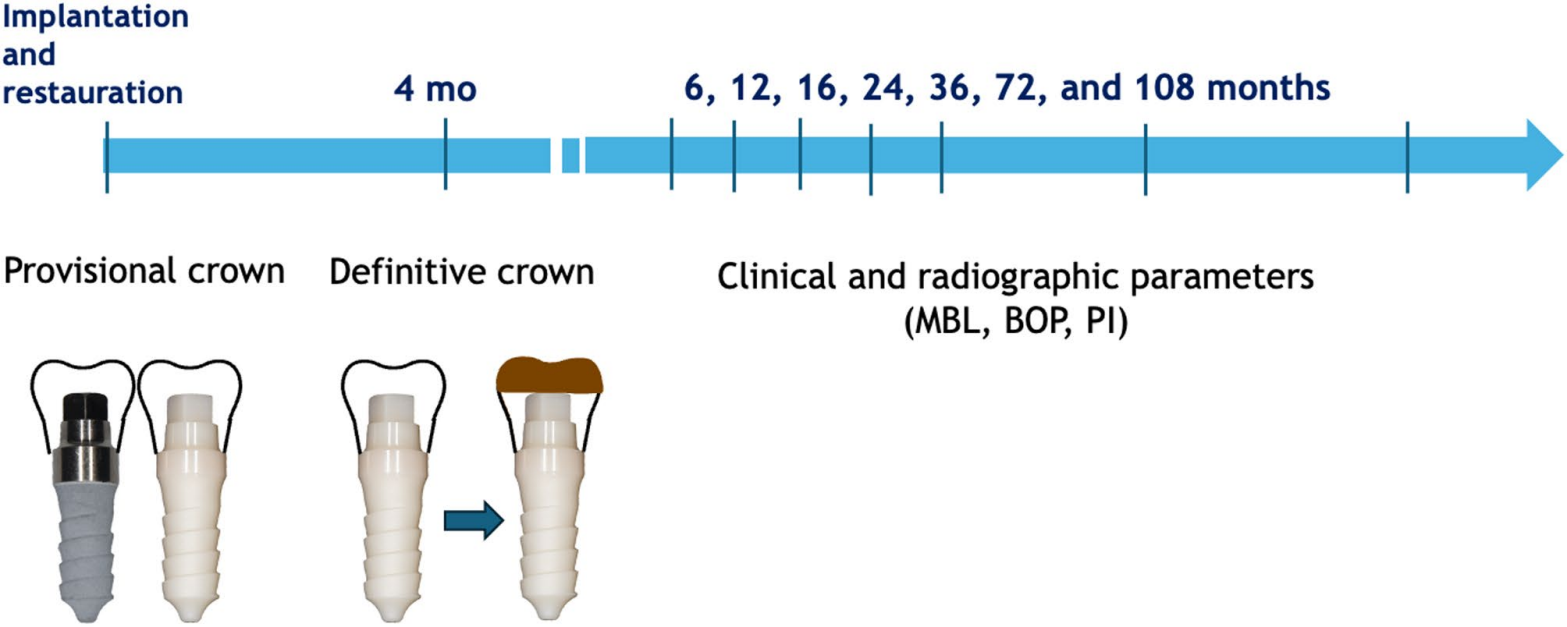
Study design and follow-ups. Directly after implant placement, all-ceramic provisional single crowns (white) were adhesively luted without centric or eccentric occlusal contacts. Following a healing period of four months, provisional crowns were reduced, and conventional impressions were taken using polyether material to fabricate the final all-ceramic crowns (brown). The final restorations were also adhesively luted to preserve the microgap-free interface between the crown and the one-piece implant

**Fig. 2:**
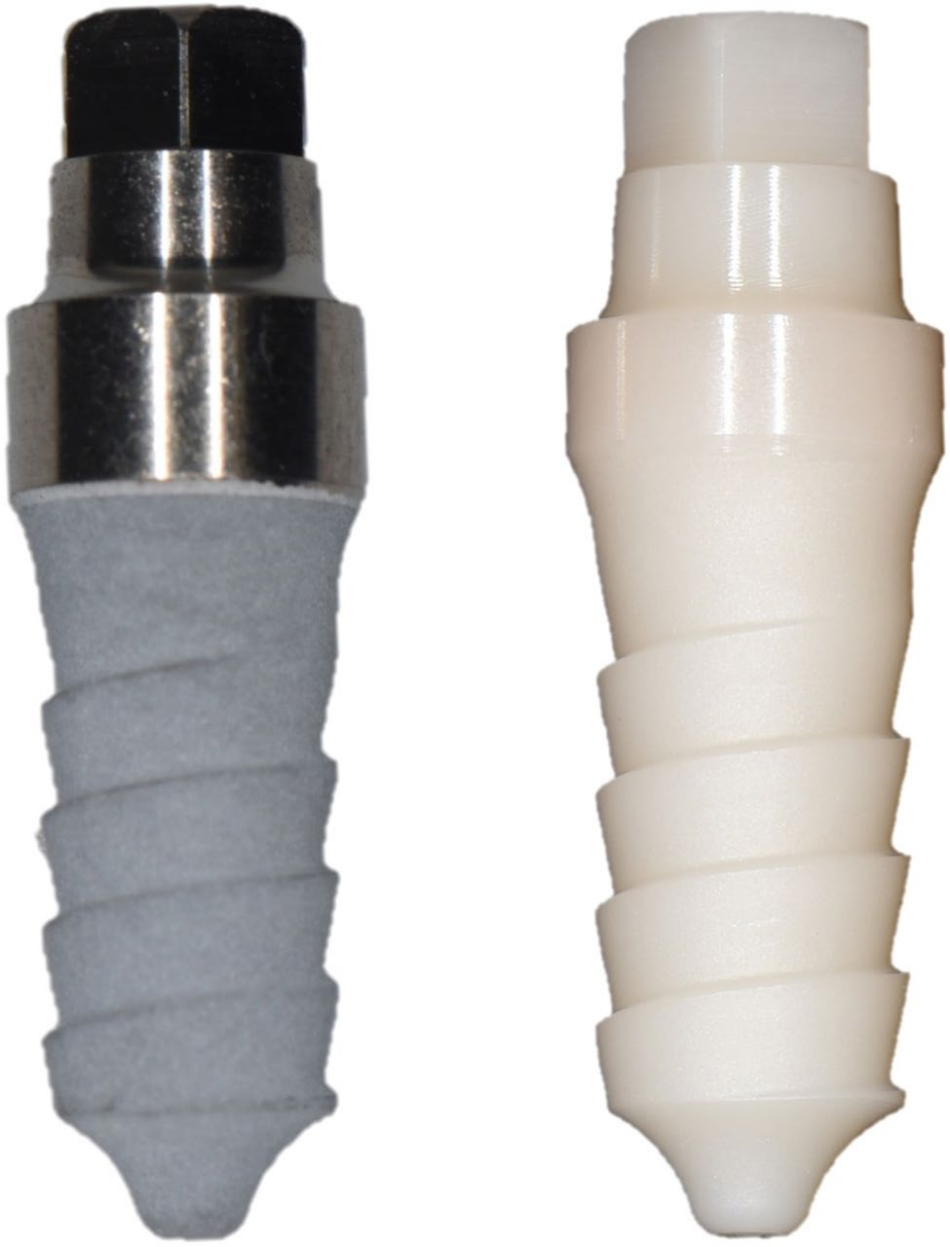
One-piece yttria-stabilised zirconia implants (Zit-Z, Ziterion GmbH, Uffenheim, Germany) with a 1.5 mm polished collar and a roughened surface (aluminum oxide blasting) and one-piece titanium implants (grade 4) (Zit-T, Ziterion GmbH, Uffenheim, Germany) with a 2.5 mm polished collar and electrochemically coated surface

Between 2010 and 2013, 22 one-piece yttria-stabilised zirconia implants (Zit-Z, Ziterion GmbH, Uffenheim, Germany) and 11 standard one-piece titanium implants (Zit-T, Ziterion GmbH, Uffenheim, Germany), with lengths ranging from 10 to 13 mm, diameters between 3.5 and 5 mm were inserted in 32 patients by three experienced surgeons of the Department of Oral Surgery and Orthodontics of the Medical University of Graz in Austria (Fig. 1). The one-piece zirconia implants were composed of Y-TZP, featuring a 1.5 mm polished transmucosal collar and an endosseous portion that was surface-treated with aluminum oxide wet shot blasting to enhance roughness. The one-piece titanium implants were manufactured from medical-grade 4 titanium, with a 2.5 mm polished transmucosal collar, and their surface was modified using an electrochemical coating process. The standardized prosthetic abutment had a height of 4 mm for all implants (Fig. 2).

Before implantation, panoramic radiography (Sidexis Intraoral, Orthophos plus DS, Sirona Dental Systems, Bensheim, Germany) was performed to evaluate the amount of bone height. An insufficient amount of bone at the implant site, as detected on the radiographs, was considered an exclusion criterion. In all participants, zirconia and titanium implants were placed no earlier than 6 months after tooth extraction (late implant insertion).

During the surgery, after local articaine infiltration anaesthesia (Ultracain Dental Forte, Sanofi-Aventis, Austria) and a mid-crestal incision, a full-thickness flap was conducted. Drilling sequences using all available diameter drills were performed according to the manufacturer’s instructions. Insertion torque was measured using a torque control device (W&H Implantmed; W&H Dentalwerk Bürmoos GmbH, Bürmoos, Austria). Implants were inserted with a terminal insertion torque greater than 30 Ncm. Immediately after implant placement, digital impressions were obtained using a CAD/CAM system (Cerec 3D, Dentsply Sirona, Bensheim, Germany) by a single prosthodontist. A clinical spectrophotometre (Easyshade Vita Zahnfabrik, Bad Säckingen, Germany) was used to determine the ceramic colour selection. Provisional crowns, made of monolithic lithium disilicate (e.max CAD-Blocks, Ivoclar-Vivadent, Schaan, Liechtenstein), were fabricated based on the digital impression using the Cerec 3D system (Cerec 3D, DentsplySirona, Bensheim, Germany). All-ceramic provisional single crowns were then constructed without centric or eccentric occlusal contacts and adhesively luted (Multilink-Automix, Ivoclar-Vivadent, Schaan, Liechtenstein) using a rubber dam. Following a healing period of four months, provisional crowns were reduced paramarginally, ensuring no interference with the submucosal peri-implant tissues. These provisional crowns served as a temporary restoration and were retained during the healing period to allow for soft tissue adaptation, before being replaced with definitive restorations. Conventional impressions were taken using polyether material (Impregum NF®, 3 M ESPE, Seefeld, Germany) to fabricate the final all-ceramic crowns. The definitive restorations were also adhesively luted (Multilink-Automix®, Ivoclar-Vivadent) to preserve the microgap-free interface between the crown and the one-piece implant (Fig. 1).

### Clinical and radiographic assessments

This clinical trial consisted of radiographic assessments conducted at baseline (after the first crown delivery) and at 6, 12, 16, 24, 36, 72, and 108 months after implant placement as well as clinical assessments including plaque index (PI), bleeding on probing (BOP) assessed at baseline, 6, 12, 24, 36, 72, and 108 months after implant placement. Additionally, implant stability (Periotest; PTV; Medizintechnik Gulden e. K., Modautal, Germany) was clinically investigated [15]. Additionally, implant success was defined as an implant in a position supporting a prosthetic suprastructure, without peri-implant translucency, implant-induced pain, infection, or paraesthesia, without implant fracture, and with an implant stability value <  + 8 (Periotest; PTV; Medizintechnik Gulden e. K., Modautal, Germany), as previously proposed by Naert et al. [16] and Snauwaert et al. [17]. The restoration survival was defined as the absence of fractures or chipping during the follow-up period.

Intraoral digital radiographs (Sidexis Intraoral, Orthophos plus DS; DentsplySirona, Bensheim, Germany) were obtained using a rectangular collimation technique with a Sirona XG AimRight sensor holder system (DentsplySirona, Bensheim, Germany) at baseline and follow-up examinations. We measured mesial and distal bone loss around each implant, defined as the marginal bone level (MBL). The MBL was determined using the implant diameter as a reference. The distance from the implant shoulder to the mesial and distal crestal bones was measured to assess MBL. If there was an overlap between the buccal and lingual bone contours, an average value was calculated. MBL is expressed as mean ± standard deviation (SD).

### Statistical analysis

Radiographic parameters and clinical parameters (BOP, PI, and MBL) at different time points (baseline and 6, 12, 16, 24, 72, and 108 months after implant placement and baseline and 6, 12, 24, 72, and 108 months after implant placement, respectively) were conducted using Mann–Whitney U test. Statistical significance was set at *p* < 0.05. Kaplan–Meier curves were utilised to determine the survival rates of zirconia and titanium implants. No statistical comparison between survival curves was conducted due to the small and unequal group sizes. Therefore, the survival curves are presented for descriptive purposes, to illustrate the overall trends observed within the cohort.

## Results

### Patient demographics

A total of 32 patients were enrolled in the study (15 males and 17 females aged 20–62 years, with a mean age of (± SD) 34 ± 9.8 years) between July 2010 and February 2013. In this clinical trial, 22 one-piece zirconia and 11 one-piece titanium implants were used. Five of the evaluated implants (15%) were located in the maxilla, and 27 were located in the lower jaw (85%) (Table 1). Details regarding implant lengths and diameters are presented in the (Table 2).

**Table 1:** Implant location

Regio	Material	Number of implants
Maxillary incisors	Zirconia	0
	Titanium	1
Maxillary premolars	Zirconia	2
	Titanium	1
Maxillary molars	Zirconia	0
	Titanium	2
Mandibular premolars	Zirconia	0
	Titanium	2
Mandibular molars	Zirconia	20
	Titanium	5

**Table 2:** Distribution of the placed implants (n) and lost implants (n) including implant length (mm) and diameter (mm)

	Implant length (mm)	Implant diameter (mm)		
		**3.5**	**4**	**5**
Placed zirconia implants	11.5	2	13	1
	13	–	6	–
Placed titanium implants	10	–	–	1
	11.5	–	7	–
	13	1	2	–
Lost zirconia implants	11.5	–	7	–
	13	–	1	–
Lost titanium implants	10	–	–	–
	11.5	–	2	–
	13	–	–	–

### Survival and success

The final insertion torque values ranged from 30 to 45 Ncm across all implants. No implant fractures were reported. In the titanium group, two implant failures (18.2%) were observed (Table 2). Two titanium implants, measuring 11.5 mm in length and 4 mm in diameter, failed after 11 months in a 48-year-old female patient and after 67 months in a 30-year-old female participant. In the one-piece zirconia implant group, a total of eight implants (36.4%) were declared failures after a mean in situ duration of (± SD) 9.9 ± 13.1 months. Two zirconia implants failed after two months, one after four months, two after five months, one after six months, one after 14 months and another one after 41 months. Most of the lost zirconia implants (85.7%) had a diameter of 4 mm and a length of 11.5 mm. The failed zirconia implants exhibited circumferential peri-implant translucency and were clinically mobile. They were removed atraumatically using forceps without surgical intervention, as they had lost osseointegration. Implant stability was assessed using the Periotest device, which measures micromobility. A Periotest Value (PTV) greater than + 8 was considered indicative of failure. Of the remaining 23 implants included in this long-term follow-up cohort study, 14 were observed over the entire 108-month period, including 8 titanium implants and 6 zirconia implants. The remaining patients were unable to attend due to personal reasons or logistical challenges.

**Fig. 3:**
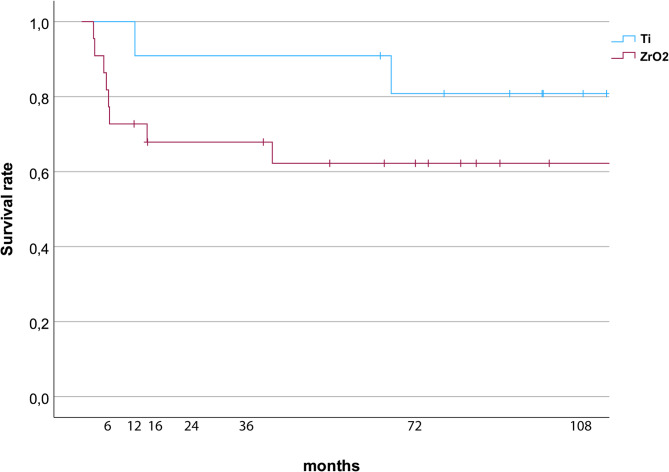
Kaplan–Meier curves exhibiting the survival probability of titanium and zirconia implants

Zirconia implant survival was 62.2% after an observation period of 108 months. A Kaplan–Meier analysis showed a survival rate of titanium implant of 80.8% up to 108 months (Fig. 3).

All restorations demonstrated 100% survival, with no complications (chipping, fracture) observed throughout the follow-up period.

### Clinical assessments

**Fig. 4:**
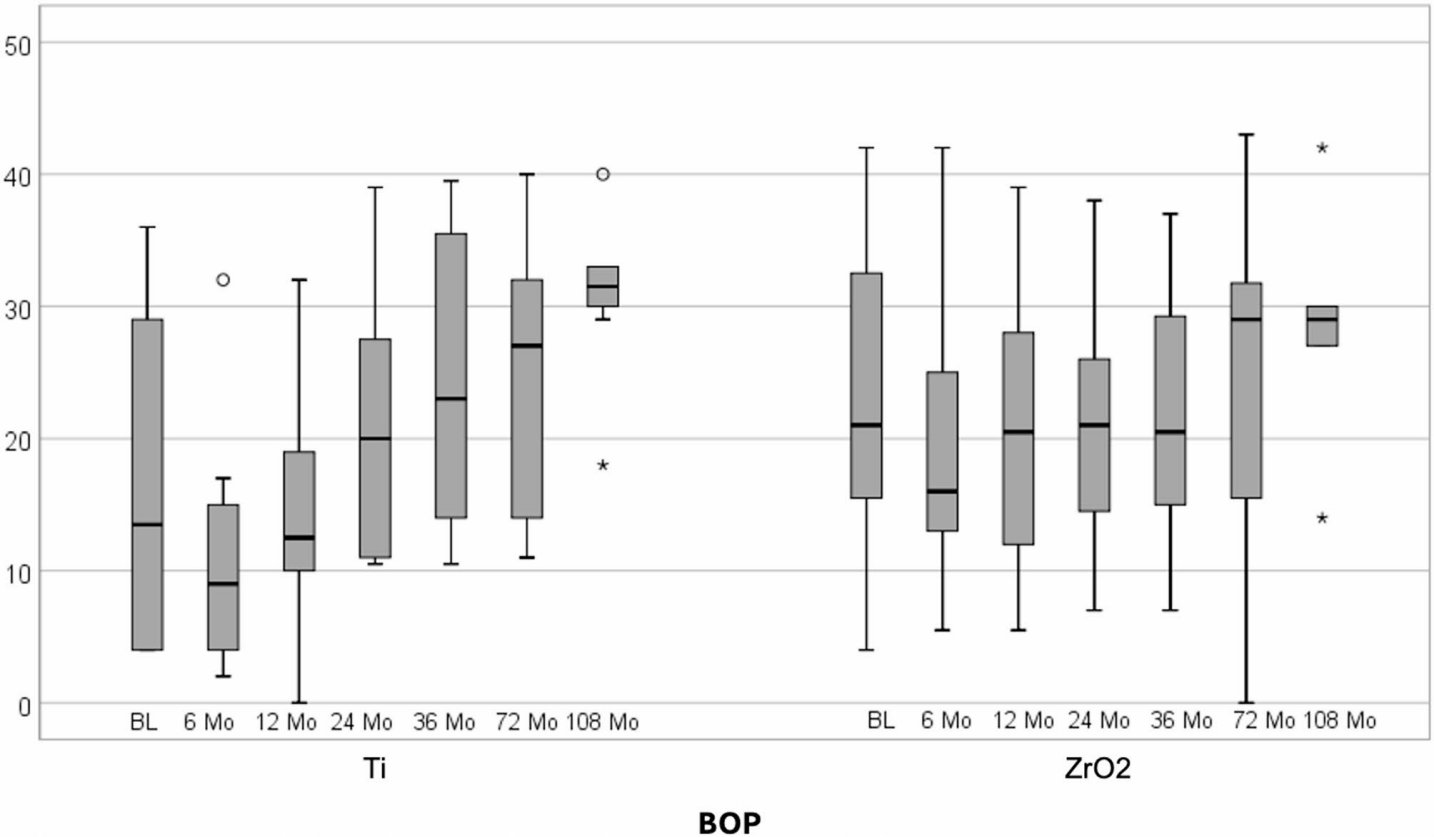
Box-plot diagram of bleeding on probing (BOP) scores at baseline, 6, 12, 24, 36, 72, and 108 months after follow-up

The mean BOP values at follow ups were 16.2% (SD ± 12.4), 10.8% (SD ± 9.0), 13.8% (SD ± 8.5), 21.0% (SD ± 10.2), 23.5% (SD ± 11.1), 25.2% (SD ± 10.3) and 30.8% (SD ± 6.1) at baseline, 6, 12, 24, 36, 72 and 108 months after titanium implant placement, respectively. For zirconia implants, the mean BOP values were 22.4% (SD ± 12.3), 19.3% (SD ± 10.0), 20.2% (SD ± 10.0), 21.4% (SD ± 9.0), 21.9% (SD ± 10.0), 23.6% (SD ± 13.6) and 28.4% (SD ± 10.0) at baseline, 6, 12, 24, 36, 72 and 108 months, respectively (Fig. 4). The mean BOP values gradually increased over time in both groups. No statistically significant difference between the two groups was observed over time (*p* > 0.05).

**Fig. 5:**
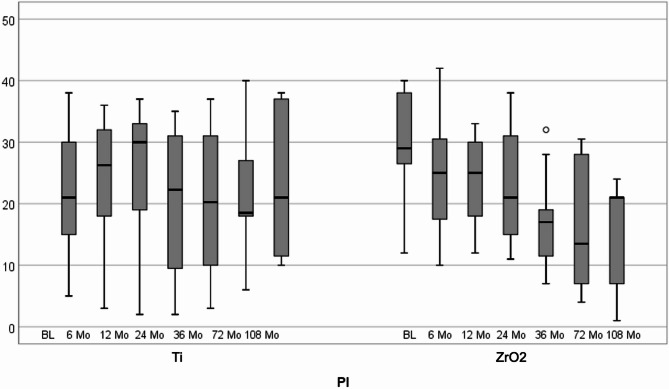
Box-plot diagram of plaque index (PI) scores at baseline and 6, 12, 24, 36, 72, and 108 months after follow-up

The PI values after titanium implant insertion revealed a mean of 21.6% (SD ± 9.9), 23.2% (SD ± 11.9), 25.7% (SD ± 10.9), 20.8% (SD ± 12.0), 20.5% (SD ± 11.7), 22.1% (SD ± 11.9) and 23.3% (SD ± 12.3) at baseline, 6-, 12-, 24-, 36-, 72- and 108-month follow-ups, respectively. After zirconia implant placement, the mean PI values were 29.2% (SD ± 9.4), 24.5% (SD ± 9.2), 24.2% (SD ± 6.8), 22.3% (SD ± 7.9), 16.6% (SD ± 7.9), 16.0% (SD ± 10.1) and 14.8% (SD ± 10.1) at baseline, 6, 12, 24, 36, 72 and 108 months, respectively (Fig. 5). A statistically significant decrease in the PI was observed in the zirconia implants after 36 and 108 months (*p* < 0.001). No statistically significant differences were observed between the two groups over time (*p* > 0.05).

### Radiographic assessments

**Fig. 6:**
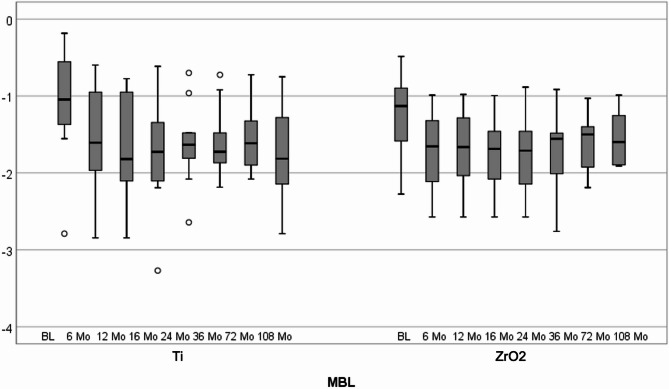
Box-plot diagram of marginal bone level (MBL) scores at baseline and 6, 12, 24, 36, 72, and 108 months after titanium and zirconia implant placement

**Fig. 7:**
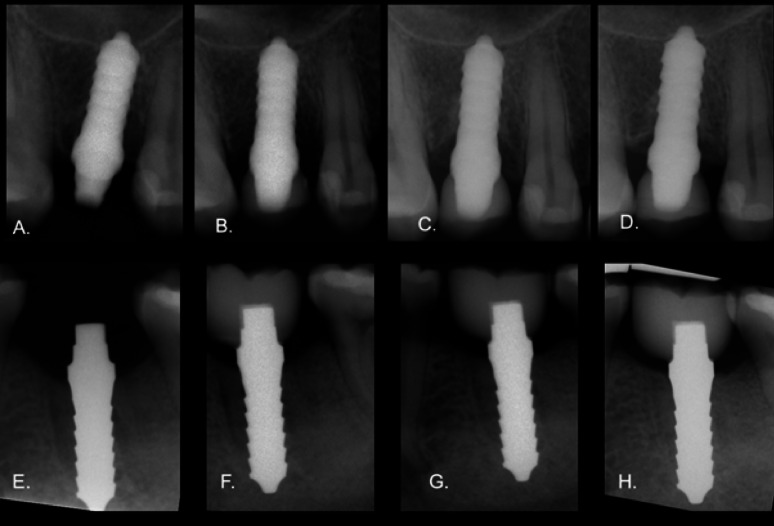
Intraoral radiographs of a one-piece titanium implant placed in tooth 14 in a 25 year-old female patient, taken directly after implantation (**A**), at baseline (**B**), 6 months (**C**), and 36 months (**D**) after implant placement. Additionally, a zirconia implant is placed in tooth 36 in a 34-year-old female patient, taken directly after implantation (**E**), at baseline (**F**), 12 months (**G**), and 72 months (**H**) after implant placement

At baseline, 6-, 12-, 16-, 24-, 36-, 72- and 108-month follow-ups, a mean MBL for titanium implants of 1.1 mm (SD ± 0.8), 1.6 mm (SD ± 0.7), 1.7 mm (SD ± 0.7), 1.7 mm (SD ± 0.7), 1.6 mm (SD ± 0.5), 1.6 mm (SD ± 0.5), 1.6 mm (SD ± 0.4) and 1.8 mm (SD ± 0.7), respectively, were reported (Figs. 6 and 7). In case of zirconia implants, the mean MBL values were 1.2 mm (SD ± 0.5), 1.7 mm (SD ± 0.5), 1.7 mm (SD ± 0.5), 1.8 mm (SD ± 0.5), 1.7 mm (SD ± 0.5),1.7 mm (SD ± 0.6), 1.6 mm (SD ± 0.5), and 1.5 mm (SD ± 0.3) at baseline, 6, 12, 16, 24, 36, 72 and 108 months after implant placement. A statistically significant increase in marginal bone resorption was observed from baseline to the 12-month follow-up in both the zirconia and titanium groups (*p* < 0.05). After this initial resorption, bone levels remained stable in both groups, with no further significant changes observed during the remaining follow-up period (*p* > 0.05).

## Discussion

In this study, we assessed the radiographic and clinical outcomes of one-piece titanium and zirconia implants after a 108-month follow-up period. Since the clinical and radiographic outcomes showed no statistically significant differences between zirconia and titanium one-piece implants restored with lithium disilicate crowns, the null hypothesis could not be rejected. Notably, the failure rate of zirconia implants was 36.4%, twice as high as that of titanium implants (18.2%).

Regarding the surgical protocol, the placement of one-piece implants must meet prosthodontic requirements, as the supracrestal portion of the implant is continuously aligned with the axis of the implant body. In the present study, one-piece implants were not designed to be ground intraorally, as was done in a previously published protocol using a different type of one-piece zirconia implants [18]. The increased fracture risk associated with intraoral grinding of one-piece zirconia implants has been discussed and, thus, seems unadvisable as a standard procedure in clinical practice. This risk appears to be primarily influenced by factors such as implant design, material properties, and the applied pressure and degree of water-cooling during abutment preparation. Consequently, intraoral as well as extraoral abutment modifications of one-piece zirconia implants should be avoided to minimise the risk of fracture [19, 20]. As a further development, various implant manufacturers have offered two-piece implant designs for zirconia implants in recent years, with promising results, though clinical data concerning hard and soft tissue integration remains limited [21, 22].

Survival analysis is essential for evaluating the long-term performance and predictability of dental implants. The Kaplan–Meier method, a widely utilised statistical approach in medical and dental research, facilitates the estimation of survival probabilities for implants over time [23]. Long-term studies have consistently demonstrated high survival rates for titanium implants. In a retrospective study, Buser et al. reported a 10-year implant survival rate of 98.8% for 511 titanium implants [24]. A recent meta-analysis collated data on the survival rate of dental implants over a 20-year period, revealing a notable success rate of 80% [25]. The present study demonstrated a 108-month survival rate of 80.8% for titanium implants, which is lower than that of previously published data. Kohler et al. also reported the loss of a two-piece titanium implant manufactured using the same process [21]. Notably, these implants are no longer commercially available. The observed implant failure may be attributed to specific surface modifications. Certain surface treatments such as acid etching or sandblasting can influence osseointegration and long-term stability [26]. Therefore, the surface characteristics of these implants may have contributed to the reduced survival rates.

Several studies utilising Kaplan–Meier survival analysis have reported promising survival rates for zirconia implants. A systematic review by Padhye et al. reported similar survival rates for zirconia and titanium implants in the short term (12 months of follow-up) [14]. In the present study, zirconia implants demonstrated a survival rate of 62.2% after a 108-month observation period. The majority of failed zirconia implants (85.7%) had a diameter of 4 mm and length of 11.5 mm. This low survival rate may be attributed to several factors. According to Roehling et al., commercially available zirconia implants demonstrate significantly higher survival rates than those of non-commercial zirconia implants. Non-commercial zirconia also shows high rates of early implant failure [27]. Hashim et al. reported a general tendency for early implant loss in first-generation zirconia implants, particularly when used in immediate or early loading protocols [28]. In this study, zirconia and titanium implants were immediately restored using lithium disilicate CAD/CAM provisionals. To ensure a stress-free healing period, provisional crowns were designed without centric or eccentric occlusal contact, thereby minimising functional loading. However, despite these precautions, implants were still subjected to indirect forces generated by masticatory processes and tongue movements. As demonstrated in a previous study, zirconia implant failure was not accompanied by general signs of inflammation [18]. Consequently, some authors proposed that these instances of failure may be categorised as “aseptic loosening” [8]. Potential causative factors include disintegration and premature loading. Recent improvements in the material properties and implant designs of zirconia, such as the introduction of two-piece zirconia implants, have contributed to enhanced survival rates. Two-piece zirconia implants, which allow for screw-retained restorations, offer better prosthetic versatility and improve clinical outcomes [29].

This study investigated first-generation zirconia implants, which were one-piece designs made from yttria-stabilised tetragonal zirconia polycrystals (Y-TZP). These implants were introduced as alternatives to titanium implants. Unlike titanium, which benefits from surface modifications such as acid etching and sandblasting, this first-generation zirconia implant has limited available surface treatments. Surface modification techniques such as sandblasting and acid etching have been applied to zirconia implants to improve osseointegration [10]. The implants used were not commercially available, and according to a systematic review and meta-analysis, implants that have been discontinued from the market exhibit a statistically lower survival rate than those currently available for commercial purchase [30].

The present study revealed a significant decrease in the PI of zirconia implants after 36 and 108 months (*p* < 0.001). A previously proposed theory suggesting reduced biofilm accumulation around zirconia implants may explain this clinical outcome [31]. However, no statistically significant differences in BOP and PI were observed between the zirconia and titanium implants over time (*p* > 0.05). A statistically significant increase in marginal bone resorption was observed in both groups during the first year after implant placement (*p* < 0.05). This is consistent with the typical early bone remodeling phase following implant placement, as reported in previous studies [32]. After this initial period, marginal bone levels stabilized, with no further significant changes in either group (*p* > 0.05). No statistically significant differences were observed between the two groups over time.

This study has certain limitations. It included only 22 zirconia and 11 titanium implants, which represents a relatively small and inhomogeneous sample. A larger cohort would increase statistical power and enhance the reliability of the findings. While the study provides long-term data comparing zirconia and titanium implants, its limitations must be considered when interpreting the results. As the study was exploratory in nature, no formal corrections for multiple comparisons (such as Bonferroni adjustment) were applied. The statistical analysis was intended to observe trends over time rather than establish definitive group differences. This approach increases the risk of Type I error and should be taken into account when interpreting statistical significance. Another notable limitation of the present study is the distribution of implant placement sites, with the majority of zirconia implants being positioned in the mandibular molar region. This site-specific clustering introduces a potential bias. Considering the mandibular molar area is subject to specific biomechanical conditions such as increased occlusal forces and variations in bone density. These factors may have influenced the clinical and radiographic outcomes observed. Future studies with larger sample sizes, longer follow-up periods, and more standardized protocols are needed to further validate the clinical performance and long-term success of zirconia implants. Future research involving a larger patient sample, randomized and controlled study, longer follow-up periods, and more standardised methodologies is needed to further validate the clinical performance and long-term success of zirconia implants.

## Conclusions

Within the limitations of this prospective cohort study, non-commercially zirconia one-piece implants demonstrated lower survival rates compared to their titanium counterparts over a follow-up period of up to 108 months. The clinical performance of the first-generation zirconia implants, now commercially unavailable, was notably inferior, aligning with outcomes reported in other clinical studies.

## Data Availability

The original data supporting the findings of this study is available from the corresponding author upon reasonable request.

## Abbreviations

BOP: Bleeding On Probing
CBCT: Cone beam computed tomography
MBL: Marginal bone level
PI: Plaque index
SD: Standard deviation
Y-TZP: Yttria-stabilised tetragonal zirconia polycrystals
